# Risk Factors of COVID-19 Patients

**DOI:** 10.1017/dmp.2021.7

**Published:** 2021-01-08

**Authors:** Ouail Ouchetto, Asmaa Drissi Bourhanbour

**Affiliations:** 1 LIMSAD-FSAC, Hassan II University of Casablanca, Casablanca, Morocco; 2 FSJES-AC, Hassan II University of Casablanca, Casablanca, Morocco; 3 Laboratory of Immunology, Ibn Rochd University Hospital Center, Casablanca, Morocco; 4 Faculty of Medicine and Pharmacy, Hassan II University of Casablanca, Casablanca, Morocco

**Keywords:** COVID-19, SARS-CoV-2, risk factors, mortality, incidence

The incidence and mortality of coronavirus disease 2019 (COVID-19) are very different between countries and continents. They seem to be low in Africa, Oceania, and some countries of Asia, but high in Western Europe and America. Many factors could have a role in this disparity, including comorbidities. The most frequent comorbidities reported in COVID-19 were advanced age, obesity, cardiovascular disease, diabetes, and cancer. Data from Italy showed that more than two-thirds of those who died of COVID-19 had diabetes.^1^ The Seattle region was among the first to report body mass index data, and a sample showed that 85% of COVID-19 patients with obesity required mechanical ventilation and 62% of them died.^2^ In a Chinese report from 138 hospitalized COVID-19 patients, 14.5% had cardiovascular disease (CVD).^3^ A multi-center study including 105 patients with cancer and 536 noncancer patients infected with COVID-19 showed that COVID-19 patients with cancer had a higher risk in all severe outcomes.^4^

In this study, we selected 112 countries with populations of at least 2.5 million and 3 deaths per million people. The data on COVID-19 incidence and mortality are obtained from the Worldometers until July 19, 2020. The prevalence of obesity and diabetes were gathered from ProCon.org and Indexmundi.com, respectively. The incidence rates of all cancers were obtained from the Global Cancer Observatory. The prevalence of cardiovascular diseases was extracted from the Global Health Data Exchange. Finally, the prevalence of advanced age was collected from the Central Intelligence Agency (see Appendix). We used the Spearman test to assess the degree of correlation of COVID-19 incidence and mortality per 1 million people with advanced age, CVD, obesity, and cancer. All correlation coefficients were positively significant except the diabetes coefficient, which was positive but not significant (see Table 1; and Figure 1).

**Table 1.** Correlation coefficients of COVID-19 incidence and mortality with high age, obesity, diabetes, cardiovascular disease (CVD), and cancer

Correlations						
		Age (65 y and over)	Diabetes	Obesity	CVD	Cancer
Incidence per million	Correlation coefficient	0.297**	0.133	0.273**	0.338**	0.348**
	Sig. (2-tailed)	0.001	0.162	0.004	0.000	0.000
	N	112	112	112	112	112
Mortality per million	Correlation coefficient	0.427**	0.179	0.381**	0.453**	0.466**
	Sig. (2-tailed)	0.000	0.059	0.000	0.000	0.000
	N	112	112	112	112	112

**Correlation is significant at the level 0.01 (2-tailed).

**Figure 1.**
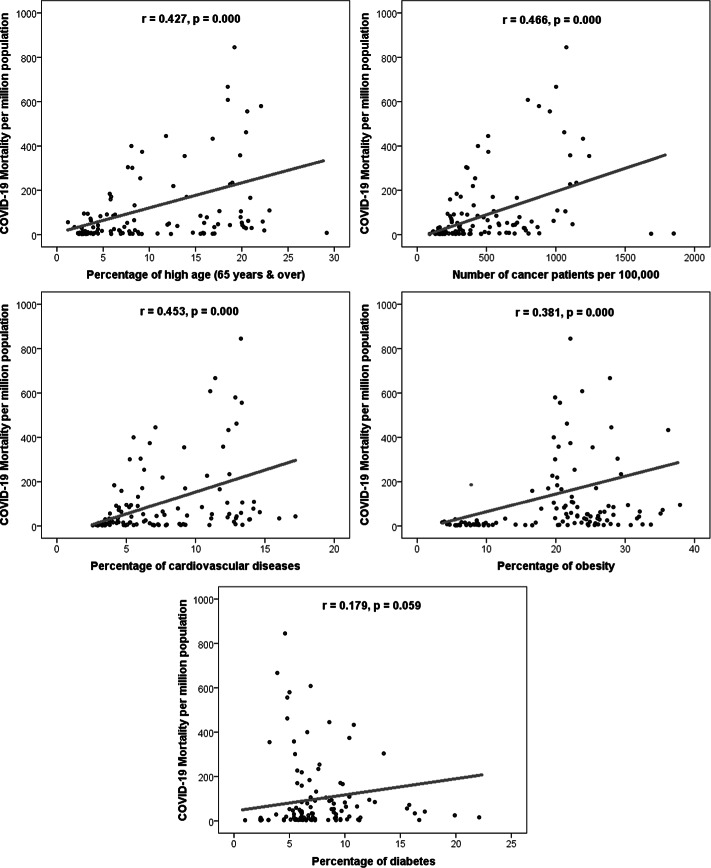
Association between COVID-19 morality per million as a function of prevalence of high age (65 y and over), number of cancer patients per 100,000, prevalence of cardiovascular diseases, prevalence of obesity, and prevalence of diabetes.

Our findings suggest that advanced age, CVD, cancer, and obesity are risk factors of COVID-19. Older people are more affected by COVID-19. This is due to the senescence of the immune system and frailty, which is a clinical syndrome characterized by an increased vulnerability to adverse health outcomes and aging-associated functional decline. CVD patients are more likely to be infected due to their deteriorated heart function. In fact, pre-existing CVD heightens the vulnerability to developing COVID-19 and to have more severe disease with worse clinical outcomes and prognosis. Cancer patients are at high risk for infection due to coexisting chronic diseases, overall weakened health status, and systemic immunosuppressive states caused by both cancer and anticancer treatments. Obesity is characterized by chronic inflammation associated with a decreased immune system, leading to susceptibility to infection. Additionally, patients with obesity show a restrictive breathing pattern and reduced lung volume. This evidence suggests that obesity may act as an independent risk.

The distinction between type 1 and 2 diabetes and the age distribution of diabetics are lacking in our study, which may explain why the correlation with diabetes was only positive but not significant. Indeed, not all diabetics are exposed to the same risks when faced with COVID-19. The study called CORONADO^5^ made it possible to profile these diabetic patients at risk. The vast majority of patients hospitalized for COVID-19 presented with type 2 diabetes.

## Supplementary material

For supplementary material accompanying this paper visit http://dx.doi.org/10.1017/dmp.2021.7. click here to view supplementary material
