# Supplementary material for: Risk Factors of COVID-19 Patients
Source: Disaster Med Public Health Prep. 2021 Jan 8:1–3. doi: 10.1017/dmp.2021.7 (PMC8007946; doi:10.1017/dmp.2021.7)
Supplement: Supplementary file 1 [file S1935789321000070sup001.docx]

**Appendix**

Table: **Worldwide cases and mortality rate of COVID-19,** number of cancer patient per 100,000, prevalence of diabetes, obesity, cardiovascular disease (CVD) and high age (65 y and over).

| Country | Deaths /  1 Million population | Incidences/  1 Million population | Cancer/  (100,000) | Diabetes  (%) | Obesity  (%) | CVD  (%) | Age 65 years & over (%) |
| --- | --- | --- | --- | --- | --- | --- | --- |
| Belgium | 845 | 5496 | 1076 | 4.6 | 22.1 | 13.27 | 19.21 |
| United Kingdom | 667 | 1346 | 1001.9 | 3.9 | 27.8 | 11.42 | 18.48 |
| Spain | 608 | 6639 | 798.2 | 6.9 | 23.8 | 11.06 | 18.49 |
| Italy | 580 | 4043 | 878.4 | 5 | 19.9 | 12.87 | 22.08 |
| Sweden | 556 | 7722 | 956.8 | 4.8 | 20.6 | 13.33 | 20.59 |
| France | 462 | 2702 | 1060.7 | 4.8 | 21.6 | 12.95 | 20.46 |
| Chile | 445 | 17304 | 512.1 | 8.6 | 28 | 7.11 | 11.81 |
| United States | 433 | 14046 | 1195.7 | 10.8 | 36.2 | 12.37 | 16.85 |
| Peru | 400 | 10717 | 438.8 | 6.6 | 19.7 | 5.52 | 8.05 |
| Brazil | 374 | 9876 | 513.3 | 10.4 | 22.1 | 6.7 | 9.21 |
| Netherlands | 358 | 3018 | 1103.4 | 5.4 | 20.4 | 11.99 | 19.82 |
| Ireland | 355 | 5214 | 1240.5 | 3.2 | 25.3 | 9.18 | 13.82 |
| Mexico | 304 | 2627 | 354.2 | 13.5 | 28.9 | 6.03 | 7.67 |
| Ecuador | 301 | 4192 | 362.9 | 5.5 | 19.9 | 5.25 | 8.15 |
| Panama | 254 | 12383 | 418.3 | 7.7 | 22.7 | 6.29 | 9.01 |
| Canada | 234 | 2922 | 1148.3 | 7.6 | 29.4 | 12.44 | 18.98 |
| Switzerland | 227 | 388 | 1102.9 | 5.7 | 19.5 | 10.83 | 18.73 |
| Armenia | 219 | 11769 | 402.2 | 6.1 | 20.2 | 7.62 | 12.6 |
| Bolivia | 184 | 4977 | 285.4 | 6.8 | 20.2 | 4.13 | 5.71 |
| Iran | 171 | 3258 | 314.2 | 9.6 | 25.8 | 6.15 | 5.87 |
| Moldova | 170 | 5202 | 545.5 | 5.7 | 18.9 | 9.25 | 14.03 |
| Portugal | 166 | 4771 | 719.2 | 9.8 | 20.8 | 11.74 | 20.92 |
| Kyrgyzstan | 159 | 4064 | 237.9 | 6.1 | 16.6 | 4.65 | 5.8 |
| Colombia | 132 | 3875 | 407.8 | 7.4 | 22.3 | 5.84 | 8.39 |
| Germany | 109 | 2421 | 1010.9 | 10.4 | 22.3 | 14.21 | 22.99 |
| Romania | 106 | 1948 | 552.6 | 6.9 | 22.5 | 13.37 | 17.58 |
| Denmark | 105 | 2274 | 1068.9 | 8.3 | 19.7 | 12.29 | 19.91 |
| Kuwait | 95 | 13854 | 321.8 | 12.2 | 37.9 | 5.01 | 2.92 |
| Iraq | 94 | 2298 | 219.1 | 8.8 | 30.4 | 5.34 | 3.33 |
| Honduras | 91 | 3308 | 264.7 | 7.3 | 21.4 | 4.27 | 5.4 |
| Dominican Republic | 90 | 487 | 361.5 | 8.6 | 27.6 | 5.82 | 6.29 |
| Russian Federation | 85 | 5287 | 571.3 | 6.1 | 23.1 | 10.46 | 15.53 |
| South Africa | 85 | 196 | 436.2 | 12.7 | 28.3 | 5.21 | 6.09 |
| Guatemala | 83 | 2157 | 238.9 | 10 | 21.2 | 4.47 | 4.7 |
| Austria | 79 | 2182 | 737.9 | 6.6 | 20.1 | 8.73 | 19.87 |
| Bosnia & Herzeg. | 78 | 2543 | 479.2 | 9 | 17.9 | 14.17 | 16.22 |
| Saudi Arabia | 72 | 268 | 246.3 | 15.8 | 35.4 | 4.45 | 3.63 |
| Turkey | 65 | 2603 | 508.3 | 11.1 | 32.1 | 7.63 | 8.35 |
| Oman | 64 | 1304 | 250 | 10.1 | 27 | 4.64 | 3.68 |
| Hungary | 62 | 449 | 984 | 6.9 | 26.4 | 14.63 | 20.69 |
| Finland | 59 | 1324 | 825.6 | 5.6 | 22.2 | 13.28 | 22.26 |
| Qatar | 56 | 37983 | 250.3 | 15.6 | 35.1 | 3.82 | 1.19 |
| Serbia | 54 | 526 | 721.2 | 9 | 21.5 | 12.43 | 20 |
| Belarus | 53 | 6995 | 649 | 5 | 24.5 | 11.18 | 15.93 |
| El Salvador | 53 | 1826 | 290.3 | 8.8 | 24.6 | 6.22 | 7.6 |
| Argentina | 50 | 2803 | 587.1 | 5.9 | 28.3 | 7.76 | 12.13 |
| Norway | 47 | 1665 | 1120.3 | 5.3 | 23.1 | 9.39 | 17.43 |
| Israel | 45 | 5468 | 706.4 | 9.7 | 26.1 | 7.19 | 11.96 |
| Bulgaria | 43 | 1257 | 591.8 | 6 | 25 | 17.2 | 20.06 |
| Poland | 43 | 106 | 691.5 | 6.1 | 23.1 | 12.96 | 18.72 |
| Egypt | 42 | 857 | 308.3 | 17.2 | 32 | 5.37 | 4.44 |
| Albania | 39 | 1421 | 401.1 | 9 | 21.7 | 12.31 | 13.03 |
| Azerbaijan | 35 | 2713 | 228.1 | 6.1 | 19.9 | 12.91 | 7.29 |
| Ukraine | 34 | 5752 | 524.5 | 6.1 | 24.1 | 11.13 | 17.03 |
| UAE | 34 | 4341 | 303.1 | 16.3 | 31.7 | 4.39 | 1.9 |
| Czechia | 34 | 1302 | 868.2 | 7 | 26 | 16.03 | 20.23 |
| Mauritania | 33 | 1262 | 161.8 | 7.1 | 12.7 | 3.77 | 3.92 |
| Croatia | 30 | 1059 | 769.8 | 5.4 | 24.4 | 13.92 | 21.06 |
| Afghanistan | 30 | 910 | 151 | 9.2 | 5.5 | 3.48 | 2.68 |
| Lithuania | 29 | 710 | 775.5 | 3.8 | 26.3 | 13.87 | 20.45 |
| Pakistan | 25 | 1192 | 200.3 | 19.9 | 8.6 | 3.94 | 4.44 |
| Algeria | 25 | 526 | 312.6 | 6.7 | 27.4 | 6.4 | 6.17 |
| Kazakhstan | 20 | 3744 | 384.4 | 6.1 | 21 | 6.27 | 8.43 |
| India | 20 | 810 | 173.5 | 10.4 | 3.9 | 4.37 | 6.72 |
| Greece | 19 | 385 | 783.8 | 4.7 | 24.9 | 12.77 | 22.43 |
| Philippines | 17 | 614 | 330.3 | 7.1 | 6.4 | 4.44 | 4.86 |
| Bangladesh | 16 | 1241 | 168.3 | 9.2 | 3.6 | 5.29 | 6.82 |
| Sudan | 16 | 250 | 158.4 | 22.1 | 8.6 | 4.05 | 3.03 |
| Nicaragua | 15 | 475 | 277.5 | 11.4 | 23.7 | 4.78 | 5.82 |
| Indonesia | 15 | 316 | 298 | 6.3 | 6.9 | 5.25 | 7.82 |
| Yemen | 15 | 54 | 115.4 | 5.4 | 17.1 | 3.77 | 2.8 |
| Cameroon | 14 | 608 | 180.5 | 6 | 11.4 | 3.02 | 3.11 |
| Liberia | 14 | 215 | 142.9 | 2.4 | 9.9 | 3.24 | 2.83 |
| Haiti | 13 | 618 | 207.3 | 6.7 | 22.7 | 4.3 | 4.33 |
| Costa Rica | 12 | 2181 | 487.4 | 9.1 | 25.7 | 6.7 | 8.76 |
| Central African Republic | 11 | 928 | 110.8 | 6 | 7.5 | 2.93 | 3.35 |
| Senegal | 10 | 7202 | 189.5 | 2.4 | 8.8 | 3.52 | 3.1 |
| Congo democratic | 9 | 477 | 153 | 6 | 9.6 | 3.47 | 2.47 |
| Uruguay | 9 | 303 | 643.2 | 7.3 | 27.9 | 8.84 | 14.71 |
| Sierra Leone | 8 | 2392 | 141 | 2.4 | 8.7 | 3.56 | 3.7 |
| Cuba | 8 | 216 | 516.8 | 9.6 | 24.6 | 9.12 | 15.8 |
| Japan | 8 | 195 | 662.4 | 5.6 | 4.3 | 12.88 | 29.18 |
| Morocco | 7 | 467 | 288.9 | 7 | 26.1 | 7.29 | 7.11 |
| Libya | 7 | 271 | 254 | 10.2 | 32.5 | 5.74 | 4.04 |
| Zambia | 7 | 171 | 266.9 | 4.5 | 8.1 | 2.9 | 2.27 |
| South Korea | 6 | 6139 | 880.7 | 6.9 | 4.7 | 7.93 | 15.92 |
| Tajikistan | 6 | 720 | 149.6 | 6.1 | 14.2 | 3.8 | 3.63 |
| Lebanon | 6 | 419 | 593.7 | 11.2 | 33.7 | 5.08 | 7.96 |
| Somalia | 6 | 362 | 148.8 | 5.1 | 8.3 | 3.04 | 2.27 |
| Mali | 6 | 122 | 202.3 | 2.4 | 8.6 | 2.9 | 3.02 |
| Slovakia | 5 | 8186 | 803.3 | 6.5 | 20.5 | 13.38 | 17.05 |
| Ghana | 5 | 890 | 228.5 | 2.5 | 10.9 | 3.81 | 4.44 |
| Australia | 5 | 463 | 1849.8 | 5.6 | 29 | 9.27 | 15.88 |
| Singapore | 5 | 214 | 703 | 5.5 | 6.1 | 6.91 | 10.89 |
| Chad | 5 | 54 | 128.9 | 6 | 6.1 | 2.81 | 2.43 |
| Paraguay | 4 | 521 | 385.4 | 9.6 | 20.3 | 5.53 | 7.88 |
| Venezuela | 4 | 418 | 461.1 | 7 | 25.6 | 4.81 | 8.18 |
| New Zealand | 4 | 310 | 1686.8 | 6.2 | 30.8 | 8.94 | 15.54 |
| Malaysia | 4 | 271 | 337.2 | 16.7 | 15.6 | 6.99 | 6.9 |
| Georgia | 4 | 258 | 329.7 | 5.8 | 21.7 | 11 | 16.85 |
| Kenya | 4 | 248 | 302.4 | 3.1 | 7.1 | 3.42 | 3.07 |
| Nigeria | 4 | 178 | 183.2 | 3.1 | 8.9 | 2.98 | 3.3 |
| Tunisia | 4 | 116 | 256.1 | 8.5 | 26.9 | 7.35 | 8.86 |
| Ivory Coast | 3 | 535 | 166.9 | 2.4 | 10.3 | 3.25 | 2.85 |
| Guinea | 3 | 498 | 131 | 2.4 | 7.7 | 3.3 | 3.91 |
| Uzbekistan | 3 | 496 | 182.4 | 6.5 | 16.6 | 4.94 | 5.87 |
| Jamaica | 3 | 261 | 461.8 | 11.3 | 24.7 | 7.02 | 9.17 |
| Malawi | 3 | 152 | 277.7 | 4.5 | 5.8 | 3.47 | 2.68 |
| Benin | 3 | 132 | 198.1 | 1 | 9.6 | 2.92 | 2.39 |
| China | 3 | 58 | 377.6 | 9.2 | 6.2 | 7.86 | 12.34 |
| Burkina Faso | 3 | 50 | 158.1 | 7.3 | 5.6 | 2.95 | 3.16 |
| Niger | 3 | 46 | 89.1 | 2.4 | 5.5 | 2.58 | 2.68 |
